# Analysis of Circulating Microvesicles Levels and Effects of Associated Factors in Elderly Patients With Obstructive Sleep Apnea

**DOI:** 10.3389/fnagi.2021.609282

**Published:** 2021-02-24

**Authors:** Jin Tan, Huifang Xing, Sha Sha, Jinwen Li, Yuyang Miao, Qiang Zhang

**Affiliations:** ^1^Department of Geriatrics, Tianjin Medical University General Hospital, Tianjin Geriatrics Institute, Tianjin, China; ^2^Tianjin Medical University, Tianjin, China

**Keywords:** obstructive sleep apnea, microvesicles, elderly, endothelial, platelet, leukocyte

## Abstract

**Background:** The incidence of obstructive sleep apnea (OSA) in the elderly is high, and the disorder is associated with a variety of chronic diseases. Microvesicles (MVs) are extracellular vesicles secreted by various cells during stimulation or apoptosis that play an important role in the pathogenesis of OSA. However, concentrations of circulating MVs in elderly patients with OSA remain unclear.

**Methods:** Patients aged >60 years old were recruited and underwent polysomnography. Circulating plasma MV concentrations, including annexin V^+^MVs, endothelial MVs (EMVs), platelet MVs (PMVs), and leukocyte MVs (LMVs) levels, were measured using a flow cytometer with different labeling methods. Potential factors affecting the concentration of circulating MVs in elderly patients with OSA were determined *via* Spearman's correlation and multiple linear regression analysis.

**Results:** Levels of circulating MVs, including both single- (annexin V^+^MVs, CD144^+^EMVs, CD41a^+^PMVs, and CD45^+^LMVs) and dual-labeled MVs (annexin V^+^CD144^+^EMVs), were elevated in elderly patients with OSA. Circulating MVs were positively correlated with OSA severity (AHI, ODI, and SPO_2min_). To some extent, obesity affected the MV concentrations in elderly patients with OSA. In addition, age and comorbidities may be associated with MV levels, but the correlations between the MV levels and age or comorbidities were not significant.

**Conclusion:** Concentrations of circulating MVs in elderly patients with OSA are associated with the labeling method used, OSA severity, and obesity. The effects of age and comorbidities on circulating MV levels require further verification using a larger sample size.

## Introduction

Obstructive sleep apnea (OSA) is a highly prevalent sleep breathing disorder. It is characterized by recurrent, partial, or complete obstruction of the upper respiratory tract during sleep, resulting in a repeated drop in blood oxygen saturation and sleep fragmentation. Studies have shown that the prevalence of OSA increases significantly with age. The prevalence of OSA is ~2–4% middle-aged men and women (aged 30–60 years) vs. 45–62% in community-dwelling elderly individuals (aged 60+ years) (Ancoli-Israel and Ayalon, 2006). The OSA not only affects the daily life of elderly people but also leads to various comorbidities. The OSA is associated with an increased risk of endothelial dysfunction disease, such as hypertension (Hou et al., 2018), diabetes (Saad et al., 2019), coronary heart disease (Konishi et al., 2019), and stroke (Castello-Branco et al., 2020). Therefore, investigating biochemical changes associated with OSA in the elderly is important for the prevention and treatment of a variety of chronic diseases.

Microvesicles are membranous vesicles or submicrons ranging in sizes from 0.1 to 1.0 μm, released by different cell types during cell activation or apoptosis (van Niel et al., 2018). The MVs are characterized by the eversion or non-eversion of phosphatidylserine (PS) in the presence of surface antigens of the parent cells from which they originate. The PS is normally located within the cell membrane; however, in the early stage of apoptosis, the PS can be everted from the interior of the cell membrane to the outer surface of the cell membrane. Annexin V is a Ca^2+^-dependent phospholipid-binding protein that can specifically bind to PS with high affinity (Burger and Oleynik, 2017). However, annexin V has well-known off-targets beyond PS. Further, the PS density throughout the cell membrane, the proportion of PS undergoing reversal during apoptosis, and the presence of anticoagulants, may affect the binding of annexin V to PS. The concentrations of the circulating MVs derived from endothelial MVs (EMVs), platelet MVs (PMVs), and leukocyte MVs (LMVs), and annexin V^+^MVs, have been reported to be elevated in patients with endothelial dysfunction diseases, such as hypertension (Boulanger, 2010), diabetes (Giannella et al., 2017), coronary heart disease (Voukalis et al., 2019), and stroke (El-Gamal et al., 2019). Studies have shown that concentrations of MVs are also elevated in patients with OSA and that they may promote the development of coronary heart disease (Jia et al., 2017). However, existing studies on OSA and MVs tend to be focused on middle-aged people and children, and there are no studies that have focused on elderly individuals till date.

In addition, the results of existing studies on circulating MVs in patients with OSA highlight some inconsistencies between middle-aged people and children. Concentrations of EMVs, PMVs and LMVs have been shown to be significantly elevated in children with OSA, whereas only one or more types of MVs were elevated in adults with OSA. We speculate that this may be related to the research population. Moreover, the methods of labeling MVs used in prior studies were inconsistent; some used single-labeling methodology, while dual-labeling was applied in other studies. Therefore, in this study, we performed both single- and dual-labeling of multiple types of circulating MVs, such as annexin V^+^MVs, EMVs, PMVs, and LMVs, in blood samples taken from elderly patients with OSA, and analyzed assessed factors that affected MV concentrations, such as the OSA severity, age, obesity, and the presence of comorbidities.

## Materials and Methods

### Patients

**Figure 1:**
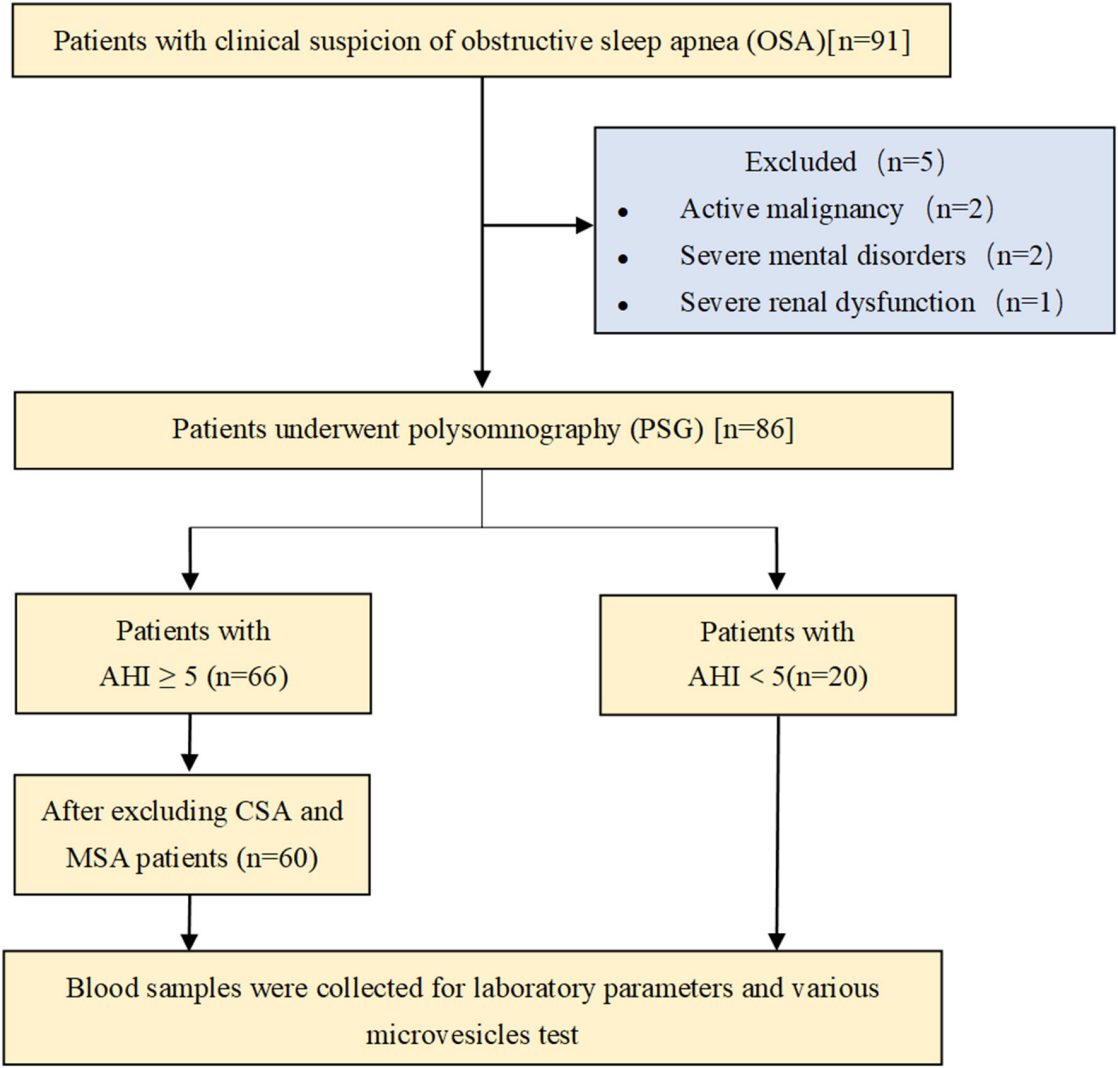
Schematic flowchart for the selection of studies.

A total of 91 patients admitted to our geriatric unit for non-acute reasons between January 2018 and September 2020 were initially enrolled in the study. Patients aged over 60 years old were included in the study. Exclusion criteria applied were active malignancy (two patients), severe mental disorder (two patients), and severe renal or liver dysfunction (one patient). Thus, 86 patients underwent polysomnography (PSG). Patients with apnea–hypopnea index (AHI) <5 events/h were included in the non-OSA (N-OSA) group (20 patients). Patients with AHI ≥ 5 events/h (66 patients) were further classified, as follows: 60 patients were included in the OSA group, and those with central or mixed sleep apnea were excluded. Blood samples were collected to assess laboratory parameters and to perform various MV tests (Figure 1). All enrolled patients signed the informed consent document approved by the Ethical Committee of Tianjin Medical University General Hospital.

### Polysomnography

Polysomnography was performed in accordance with the guidelines of the American Society of Sleep Medicine. The AHI, oxygen desaturation index (ODI) ≥3%, minimum pulse oxygen saturation (SPO_2min_), pulse oxygen mean saturation (SPO_2mean_), maximum apnea/hypopnea time (AHT_max_), and mean apnea/hypopnea time (AHT_mean_) were recorded as indices for the severity of OSA. In addition, microarousal index (MAI), total sleep time (TST), and sleep efficiency were recorded.

### Preparation of Blood Samples

Two milliliters of venous whole blood was collected from each patient (with and without OSA) in the morning following overnight PSG between 06:00 and 07:00, before breakfast. Peripheral blood was collected in acid citrate tubes and was processed within 2 h. During processing, the whole blood was centrifuged at 120 *g* for 20 min to obtain platelet-rich plasma, which was then centrifuged at 1,500 *g* for 20 min to obtain platelet-poor plasma. Further centrifugation at 13,000 *g* for 3 min produced cell-free plasma (CFP) (Yun et al., 2010). All the centrifugations described above were performed at 20°C. The CFP was then stored at −80°C until further use. Subsequently, after 2–6 weeks, frozen samples were thawed at room temperature and subsequently analyzed by a flow cytometer. All samples were frozen and thawed only once.

### Flow Cytometry Analysis of MVs

**Figure 2:**
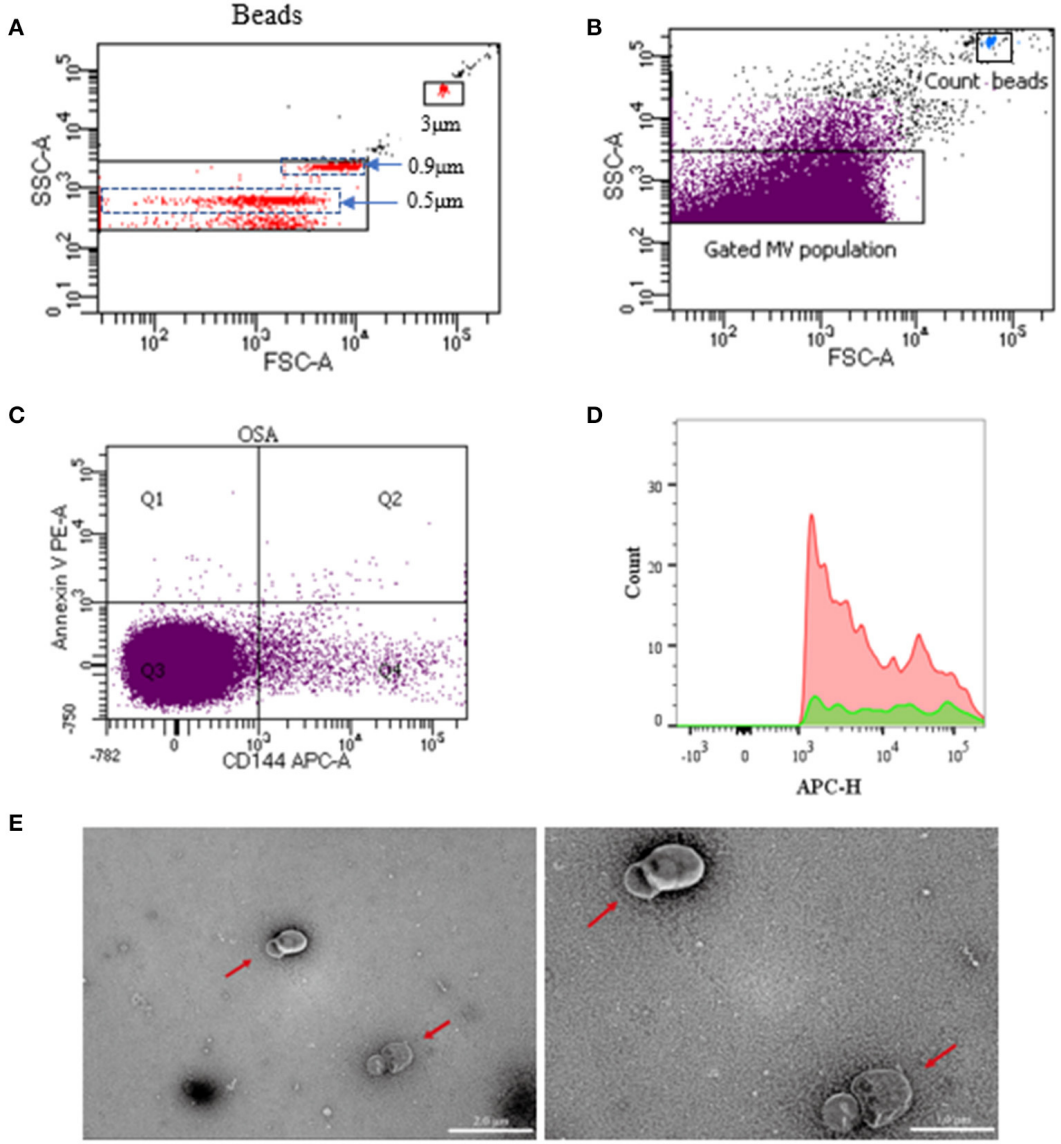
Representative dot plots of flow cytometry for circulating MVs. Overview of gating strategy of MVs in the plasma. Microbeads of 0.5, 0.9, and 3 μm in diameter were used for gating MVs. **(A)** Once the MV gate was determined, it would not be changed. Then, without microbeads, MVs in specimens were gated and analyzed. **(B)** Gated MV population in the plasma and dot plots of count beads in the sample. **(C)** Representative dot plots of dual-labeled EMVs (Section Q2). **(D)** Representative dot plots of positive single-labeled EMVs. **(E)** Transmission electron microscopy of MVs (the red arrow). The ruler at the left measures 2 μm, and the ruler at the right measures 1μm. MVs, microvesicles; EMVs, endothelial microvesicles.

Samples were analyzed using a flow cytometer (BD LSRFortessa^TM^, USA), in compliance with Food and Drug Administration performance standards and Part 15 of the rules of the Federal Communications Commission. Samples were first identified by their size (0.1–1.0 μm) using standard microbeads that measured 0.5, 0.9, and 3 μm, respectively, in diameter (Figure 2). The MV subpopulations were discriminated in the CFP according to their expression of membrane-specific antigens. The MVs derived from endothelial cells, platelets, and leukocytes were identified using anti-CD144-APC, anti-CD41a-FITC, and anti-CD45-BV650 (BD Biosciences, USA). The PE-Annexin V (BD Biosciences, USA) was used to characterize the PS-expressing MVs. Fifty microliters of CFP was incubated with 2.5 μl anti-CD144, 10 μl anti-CD41a, and 2.5 μl anti-CD45 antibodies for 30 min. Then, the mixtures were incubated with 5 μl annexin V and 50 μl 2 × annexin V binding buffer (BD Biosciences, USA) for 15 min at room temperature in darkness. After 45 min of incubation, 10 μl of count beads (Spherotech, USA) were added to the sample for calculating the absolute MV quantities, and samples were then diluted with 500 μl phosphate buffered saline. The inner and outer tubes of the cytometer were cleaned before and after each experiment. In addition, all the buffers used for the MV detection were filtered with a 0.1-μm filter to reduce non-cellular MV contamination. Debris from the flow cytometer was detected in fewer than 100 events/s when running, while about 4,000 events/s were determined for the blood sample. Sample analysis was running at low speed and terminated after a count of 60 s. The MV concentrations were calculated using the following equation:

$$\begin{array}{l} \left(\text{A}/\text{B}\right)\times \left(\text{C}/\text{D}\right) \end{array}$$

where A is the number of events for the test sample, B is the number of events for the count beads in the test, C is the number of beads per 10 μl for the lot, and D is the volume of test sample initially used.

### Transmission Electron Microscopy

Three milliliters of fresh CFP were centrifuged at 100,000 *g* for 1 h to obtain sediment, which was then resuspended with 100 μl of 0.9% saline to obtain pure MVs. Pure MVs were added to a carbon membrane/formvar membrane-coated copper mesh and incubated for 2 min at room temperature. Phosphotungstic acid (pH 6.5) was added for 2 min to enhance the visibility of the membrane structure. Then the samples were dehydrated with absolute ethanol. Digital images were acquired using a Hitachi HT-7700 transmission electron microscope (TEM, Japan). As shown in the photos of the specimens, the MVs were separated from each other and were found to be spherical or horseshoe-shaped (the red arrow in Figure 2E). The background of the field of view was also very clean, with no impurities or MV fragments.

### Data Analysis

Quantitative data are expressed as means ± standard deviation or median (inter-quartile range, IQR). An independent sample *t*-test was used to compare data from two groups, and the Mann–Whitney *U*-test was used if data were not normally distributed. A one-way ANOVA was used for comparing the data of three or more groups. Representative dot plots of positive single-labeled MVs were created using FlowJo, a flow cytometric data analysis software. Categorical data were expressed as percentages and compared using the χ^2^ test. Pearson's correlations were performed to explore the possible linear relationship between the severity of MVs and OSA. Multiple linear regression was used to analyze the influence of various parameters on MV concentrations in OSA. The values of *p* < 0.05 were considered statistically significant. All analyses were performed using SPSS 25.0 software.

## Results

### Characteristics of Cohort

Elderly patients who underwent PSG in this study were split into N-OSA (*n* = 20) and OSA (*n* = 60) groups according to AHI (Table 1). The two groups differed according to age, obesity, blood pressure, lipids, and the prevalence of hyperlipidemia. Obesity appeared more frequently in the OSA group than in the N-OSA group. Patients in the OSA group had a significantly higher mean body mass index (BMI) (*p* < 0.001), neck circumference (*p* < 0.001), and waist circumference (*p* < 0.001) than those in the N-OSA group. There were no significant differences detected with regard to sex, smoking, fasting glucose level, and heart rate between the two groups. Comorbidities, including hypertension, diabetes, coronary heart disease, stroke, and medications in the two groups were found to be similar (see Table 1).

**Table 1:** Clinical characteristics of elderly patients with and without OSA.

	**N-OSA (*n* = 20)**	**OSA (*n* = 60)**	***p*-value**
**Anthropometric characteristics**			
Age (years)	88.8 ± 8.2	81.5 ± 10.7	0.001
Sex [male (%)]	13 (65.0%)	47 (78.3%)	0.233
BMI(kg/m^2^)	22.9 ± 3.2	26.0 ± 4.7	0.004
Neck circumference(cm)	37.2 ± 3.0	40.2 ± 3.7	0.010
Waist circumference(cm)	93.8 ± 10.4	100.9 ± 9.9	0.020
Smoking [no. (%)]	7 (35.0%)	24 (40.7%)	0.653
**Laboratory parameters**			
SBP (mmHg)	135 ± 21	147 ± 20	0.022
DBP (mmHg)	69 ± 14	77 ± 12	0.080
HR (bpm)	69 ± 12	71 ± 11	0.447
TC (mmol/L)	4.83 ± 0.97	4.60 ± 0.94	0.292
HDL (mmol/L)	1.27 ± 0.38	1.10 ± 0.31	0.058
LDL (mmol/L)	2.63 ± 0.77	2.58 ± 0.73	0.757
TG (mmol/L)	1.29 ± 0.81	1.93 ± 1.34	0.054
Fasting glucose (mmol/L)	5.53 ± 0.98	5.96 ± 1.56	0.284
**Comorbidities [no. (%)]**			
Hypertension	12 (69.6%)	47 (78.3%)	0.107
Hyperlipidemia	1 (5.0%)	14 (23.7%)	0.040
Diabetes	5 (25.0%)	27 (45.0%)	0.114
Coronary heart disease	16 (80.0%)	40 (66.7%)	0.260
Stroke	4 (20.0%)	21 (35.0%)	0.330
**Medication [no. (%)]**			
Antihypertensive	13 (65%)	37 (62.7%)	0.854
Antidiabetic	3 (15%)	18 (30.5%)	0.175
Aspirin	9 (45%)	24 (40.7%)	0.735
Statins	7 (35%)	26 (48.3%)	0.303

*Data are presented as mean ± SD or no. (%). OSA, obstructive sleep apnea; N-OSA, none of obstructive sleep apnea; BMI, body mass index; SBP, systolic blood pressure; DBP, diastolic blood pressure; HR, heart rate; TC, Total cholesterol; HDL, high-density lipoprotein; LDL, low-density lipoprotein; TG, triglycerides*.

Median of AHI, ODI, AHT_max_, and snore values were significantly higher in the OSA group than in the N-OSA group, whereas SpO_2min_ and SpO_2mean_ were significantly lower in the OSA group than in the N-OSA group. However, there were no significant differences in median AHT_mean_, MAI, TST, and sleep efficiency values between the two groups (Table 2).

**Table 2:** Indicators of PSG in elderly patients with and without OSA.

**OSA parameters**	**N-OSA (*n* = 20)**	**OSA (*n* = 60)**	***p*-value**
AHI (events per hour)	1.3 (0.6–3.1)	22.5 (9.1–32.8)	<0.001
ODI (events per hour)	1.5 (0.2–3.2)	18.8 (11.1–35.4)	<0.001
SpO_2min_ (%)	88.0 (85.0–91.0)	80.5 (67.5–87.0)	<0.001
SpO_2mean_ (%)	95.0 (92.0–97.0)	93.0 (91.0–95.0)	0.008
AHT_max_ (s)	39.5 (25.5–58.0)	59.0 (47.5–80.9)	0.002
AHT_mean_ (s)	20.6 (17.8–20.9)	25.6 (19.9–30.7)	0.465
MAI (events per hour)	6.8 (3.2–13.2)	10.5 (7.0–20.6)	0.076
Snore (times)	0 (0–19.5)	32 (2.5–129)	0.006
TST (min)	415 (317–473)	371 (303–432)	0.201
Sleep efficiency (%)	82.8 (71.2–87.6)	75 (67.5–87.0)	0.902

*Data are presented as median (IQR). IQR, Inter-Quartile Range; PSG, polysomnography; AHI, apnea-hypopnea index; ODI, oxyhemoglobin desaturation index; SpO_2min_, minimum pulse oxygen saturation; SpO_2mean_, mean pulse oxygen saturation; AHT_max_, maximum apnea/hypopnea time; AHT_mean_, mean apnea/hypopnea time; MAI, microarousal index; TST, total sleep time*.

### Circulating MV Types in Elderly Patients With and Without OSA

**Figure 3:**
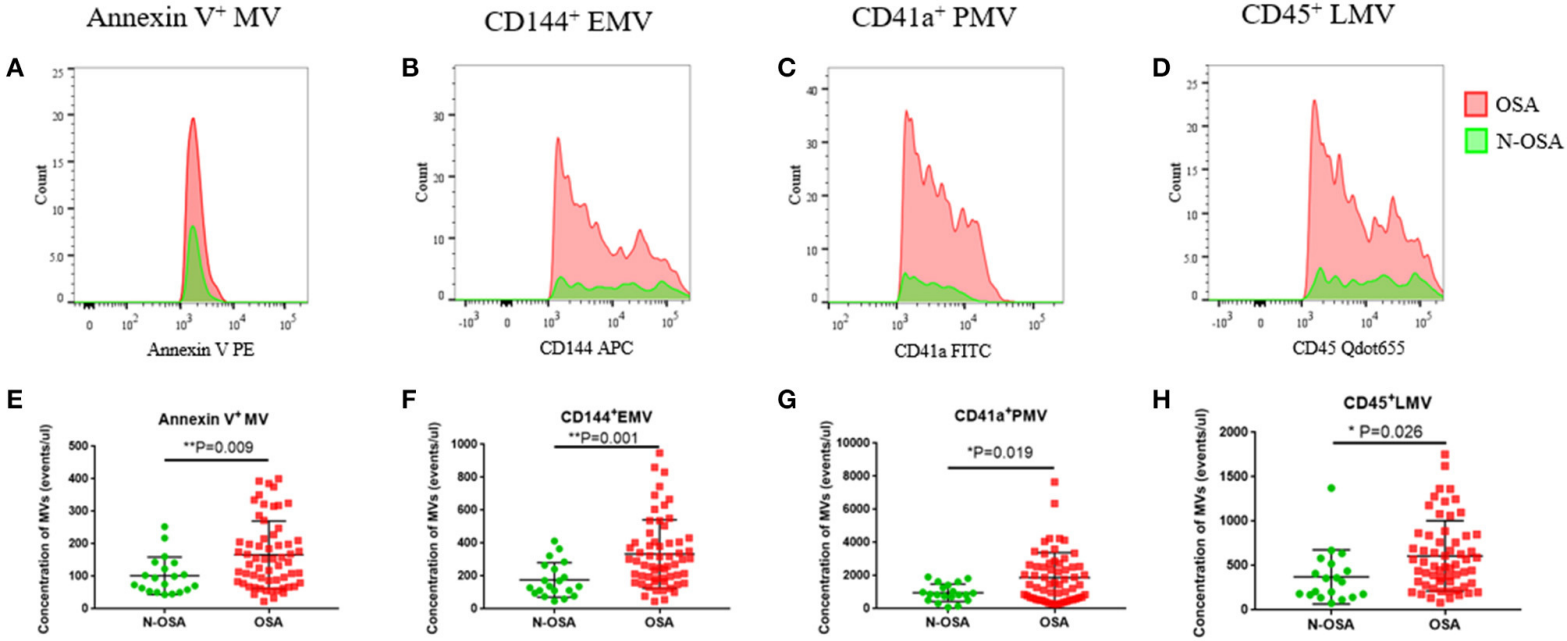
Single-labeled MVs in elderly patients with and without OSA. **(A)** Histogram of positive single-labeled annexin V^+^ MVs; **(B)** EMVs; **(C)** PMVs; **(D)** LMVs; **(E)** statistical results of annexin V^+^ MVs; **(F)** EMVs; **(G)** PMVs; **(H)** LMVs in elderly patients with and without OSA. MVs, microvesicles; OSA, obstructive sleep apnea; EMVs, endothelial microvesicles; PMVs, platelet microvesicles; LMVs, leukocyte microvesicles.

**Figure 4:**
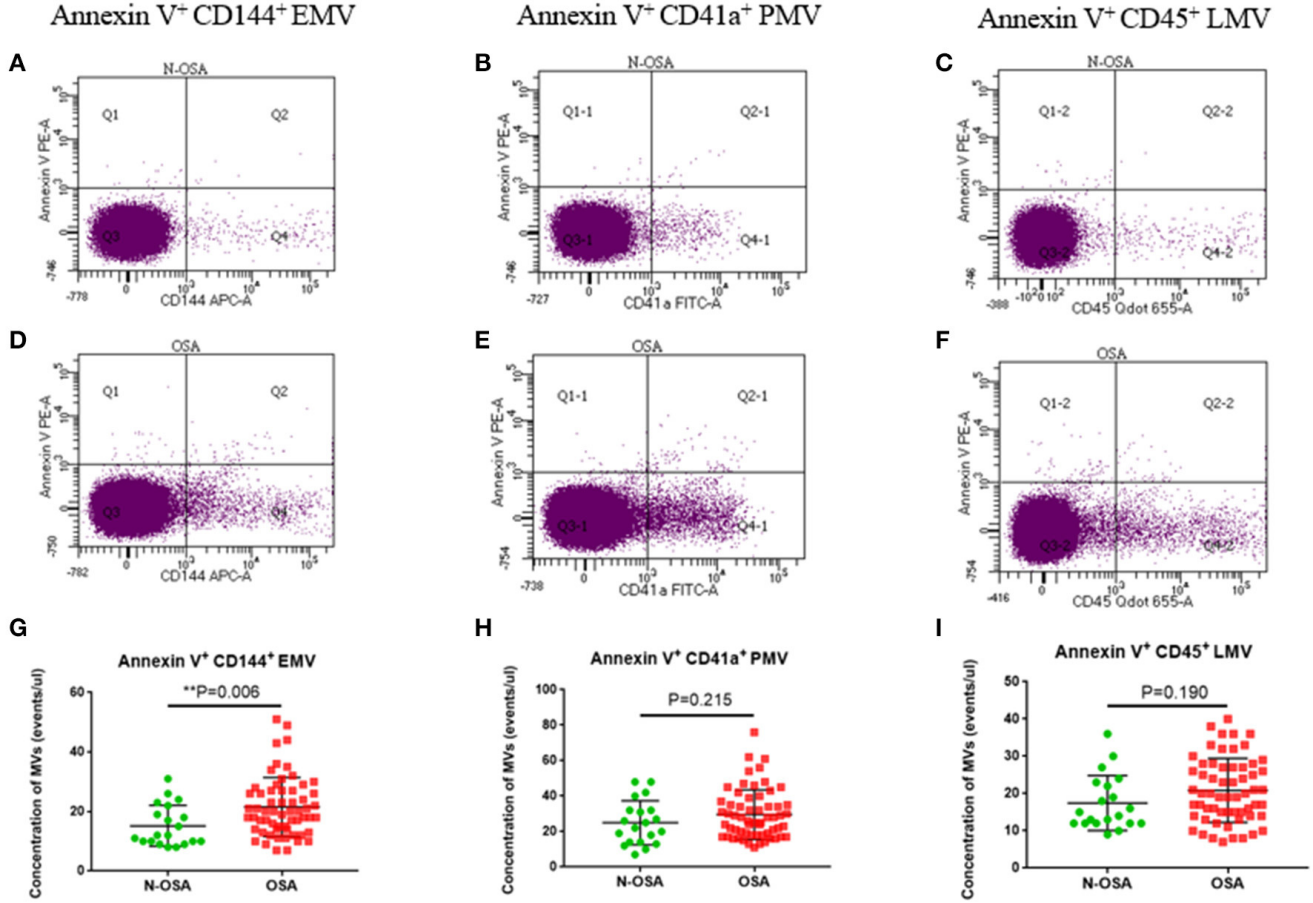
Dual-labeled MVs in elderly patients with and without OSA. Dot plots of dual-labeled **(A)** EMVs; **(B)** PMVs; **(C)** LMV in elderly patients without OSA; **(D)** dual-labeled EMVs; **(E)** PMVs; **(F)** LMVs in elderly patients with OSA; **(G)** statistical results of EMVs; **(H)** PMVs; **(I)** LMVs compared between the elderly OSA group and the elderly N-OSA group. MVs, microvesicles; OSA, obstructive sleep apnea; EMVs, endothelial microvesicles; PMVs, platelet microvesicles; LMVs, leukocyte microvesicles.

We measured concentrations of circulating MV types in patients with and without OSA *via* both single-labeling of MVs (annexin V^+^MVs, CD144^+^EMVs, CD41a^+^PMVs, and CD45^+^LMVs) and dual-labeling of MVs (annexin V^+^CD144^+^EMVs, annexin V^+^CD41a^+^PMVs, and annexin V^+^CD45^+^LMVs), and findings from these tests are shown in Table 3. We found that the concentrations of all single-labeled MV subtypes were increased significantly in elderly patients with OSA vs. elderly patients without OSA (Figure 3). In the dual-labeled MV types, annexin V^+^CD144^+^EMVs alone were increased significantly in the OSA group. Although annexin V^+^CD41a^+^PMVs and annexin V^+^CD45^+^LMVs tended to be increased in elderly patients with OSA, differences between the groups assessed were not significant (Figure 4).

**Table 3:** Circulating MV types in elderly patients with and without OSA.

**Origin**	**N-OSA (*n* = 20)**	**OSA (*n* = 60)**	***p*-value**
Annexin V^+^	86 (53–137)	148 (81–214)	0.009
**Endothelial cells**		
CD144^+^	137 (100–260)	296 (184–406)	0.001
Annexin V^+^ CD144^+^	9.9 (12.3–21.2)	19.8 (14.2–27.3)	0.006
**Platelets**		
CD41a^+^	862 (572–1392)	1487 (647–2628)	0.019
Annexin V^+^ CD41a^+^	23.1 (13.7–32.1)	27.0 (18.4–36.7)	0.215
**Leukocytes**		
CD45^+^	337 (173–467)	502 (307–837)	0.026
Annexin V^+^ CD45^+^	14.6 (12.1–23.1)	19.9 (14.5–27.3)	0.090

*Data are presented as median (IQR) of MV numbers in serum. MV types are defined based on events per microliter of plasma (events/μL). MV, microvesicle. See Table 1 legend for expansion of abbreviations*.

### Correlation Between MV Subtypes and Severity of OSA

We then investigated possible correlations between MV subtypes and the severity of OSA. Since the OSA and N-OSA groups differed according to AHI, ODI, SpO_2min_, SpO_2mean_, AHT_max_, and snore among recorded indicators of PSG, these indicators were used to reflect the severity of OSA. The correlation between the indicators and MV subtypes was analyzed. In general, we found that there was a positive correlation between the severity of OSA and the level of circulating MVs in the elderly patients considered. However, correlations between different OSA parameters and circulating MV types varied. Specific correlation coefficients and *p*-values are listed in Table 4.

**Table 4:** Correlation between MVs types and severity of OSA.

**MV subtypes**	**AHI**		**ODI**		**SPO**_****2min****_		**SPO**_****2mean****_		**AHT**_****max****_		**Snore**	
	***r***	***p*-value**	***r***	***p*-value**	***r***	***p*-value**	***r***	***p*-value**	***r***	***p-*value**	***r***	***p*-value**
Annexin V^+^ MV	0.312	0.005	0.367	0.001	−0.170	0.132	−0.108	0.339	0.176	0.122	0.101	0.372
CD144^+^EMV	0.379	0.001	0.432	<0.001	−0.394	<0.001	−0.365	0.001	0.206	0.071	0.125	0.269
Annexin V^+^ CD144^+^EMV	0.454	<0.001	0.482	<0.001	−0.410	<0.001	−0.366	0.001	0.354	0.001	0.008	0.945
CD41a^+^PMV	0.302	0.007	0.341	0.002	−0.274	0.014	−0.204	0.069	0.199	0.081	0.215	0.056
Annexin V^+^ CD41a^+^PMV	0.356	0.001	0.342	0.002	−0.377	0.001	−0.313	0.005	0.382	0.001	0.095	0.402
CD45^+^LMV	0.348	0.002	0.340	0.002	−0.346	0.002	−0.217	0.054	0.275	0.015	0.150	0.284
Annexin V^+^ CD45^+^LMV	0.329	0.003	0.366	0.001	−0.302	0.006	−0.193	0.086	0.267	0.018	0.033	0.772

### Factors That May Affect the Concentration of Circulating MVs in Elderly Patients With OSA

**Figure 5:**
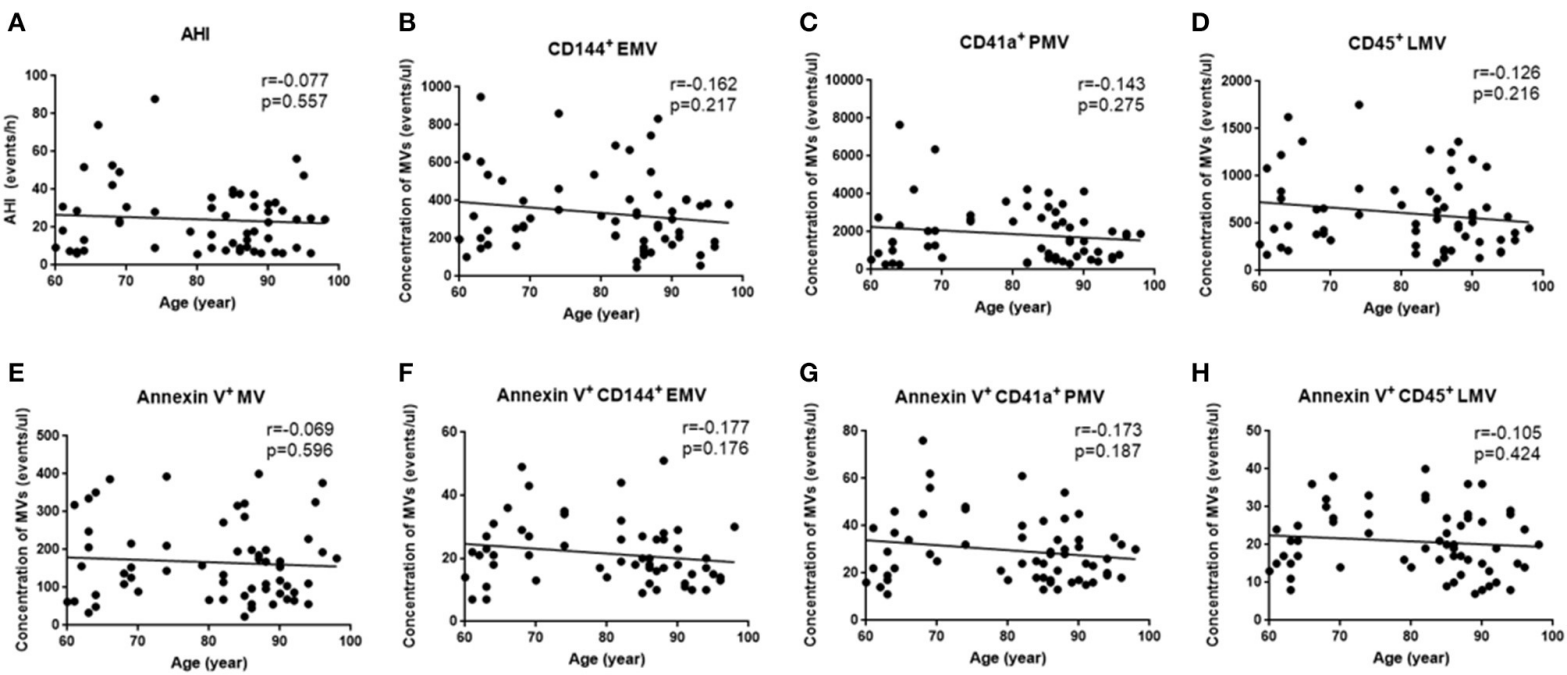
Effect of age on the concentration of MVs in elderly patients with OSA. **(A)** Correlation between age and AHI; **(B)** CD144^+^ EMVs; **(C)** CD41a^+^PMVs; **(D)** CD45^+^LMVs; **(E)** Annexin V^+^MVs; **(F)** Annexin V^+^CD144^+^ EMVs; **(G)** Annexin V^+^CD41a^+^PMVs; **(H)** Annexin V^+^CD45^+^LMVs. AHI, apnea–hypopnea index; *r*, correlation coefficient; *p, p*-value. MVs, microvesicles; OSA, obstructive sleep apnea; AHI, apnea–hypopnea index; EMV, endothelial microvesicles; PMVs, platelet microvesicles; LMVs, leukocyte microvesicles.

**Figure 6:**
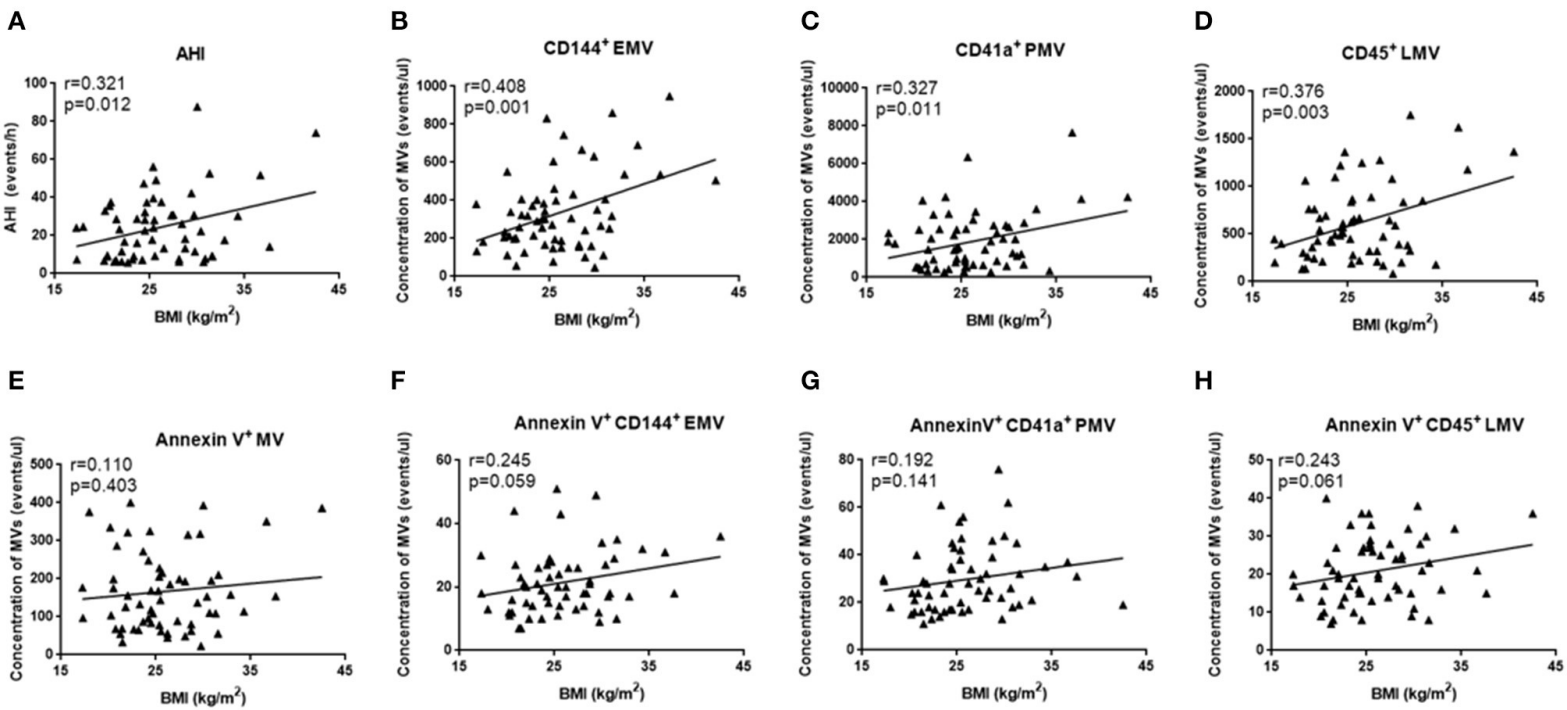
Effect of BMI on the concentration of MVs in elderly patients with OSA. **(A)** Correlation between BMI and AHI; **(B)** CD144^+^ EMVs; **(C)** CD41a^+^PMVs; **(D)** CD45^+^LMVs; **(E)** Annexin V^+^MVs; **(F)** Annexin V^+^CD144^+^ EMVs; **(G)** Annexin V^+^CD41a^+^PMVs; **(H)** Annexin V^+^CD45^+^LMVs. AHI, apnea–hypopnea index; *r*, correlation coefficient; *p, p*-value. MVs, microvesicles; OSA, obstructive sleep apnea; AHI, apnea–hypopnea index; EMVs, endothelial microvesicles; PMVs, platelet microvesicles; LMVs, leukocyte microvesicles.

**Figure 7:**
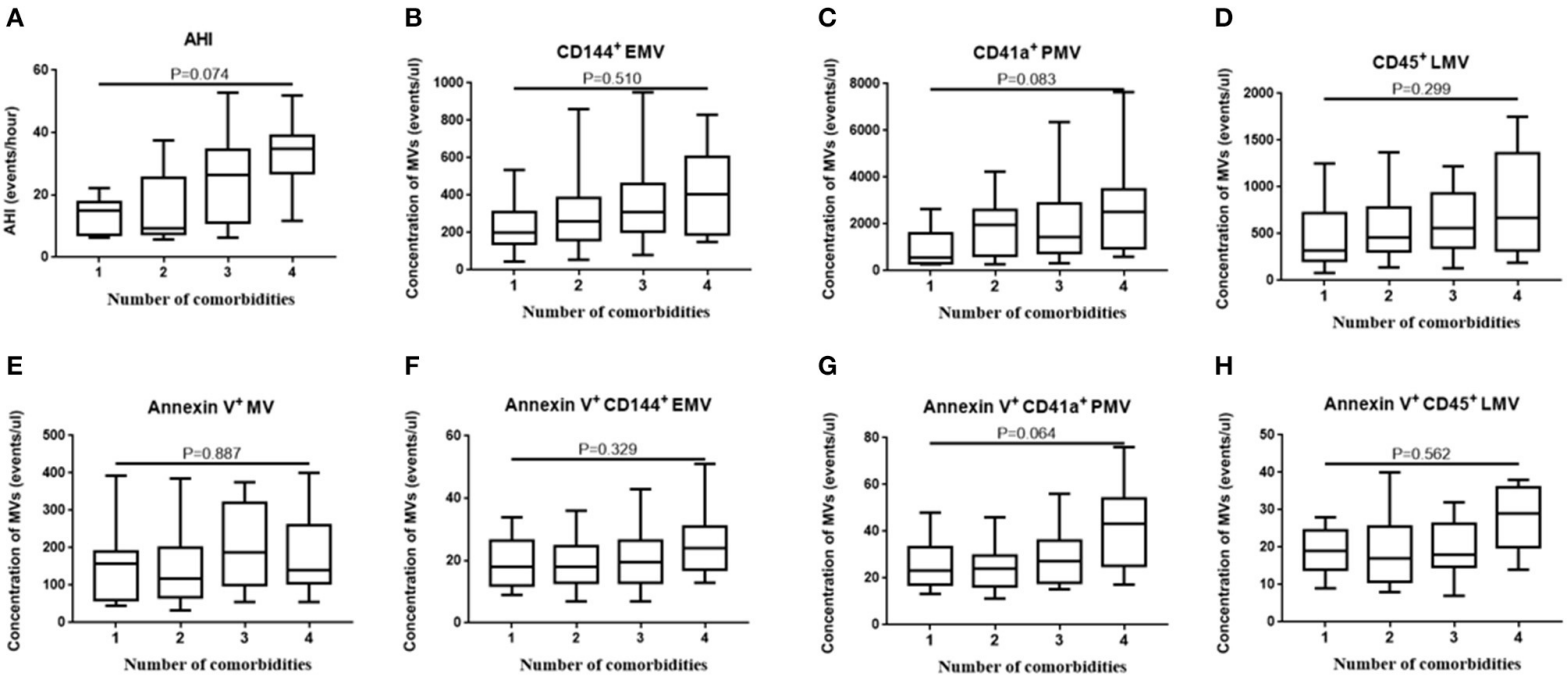
Effect of comorbidities on the concentration of MVs in elderly patients with OSA. The elderly patients with OSA were divided into 4 groups according to the number of comorbidities. **(A)** AHI; **(B)** CD144^+^ EMVs; **(C)** CD41a^+^PMVs; **(D)** CD45^+^LMVs; **(E)** Annexin V^+^MVs; **(F)** Annexin V^+^CD144^+^ EMVs; **(G)** Annexin V^+^CD41a^+^PMVs; **(H)** Annexin V^+^CD45^+^LMVs of the 4 groups were compared using one-way ANOVA. MVs, microvesicles; OSA, obstructive sleep apnea; AHI, apnea–hypopnea index; EMVs, endothelial microvesicles; PMVs, platelet microvesicles; LMVs, leukocyte microvesicles.

Since OSA and N-OSA groups differed according to age, obesity, and the presence of comorbidities, we analyzed the effects of the parameters on MV concentration. We investigated possible correlations between age, AHI, and MV subtypes, and found that the AHI decreased with the increase in age. Correspondingly, the level of each MV type showed a decreasing trend. However, there were no significant differences between age and AHI and the level of each MV type (*p* > 0.05; Figure 5). Subsequently, we investigated possible correlations between BMI, AHI, and MV subtypes, and found that AHI was positively correlated with BMI (*r* = 0.321, *p* = 0.012), and accordingly, the level of each MV type increased with increasing BMI, especially with respect to CD144^+^EMV (*r* = 0.408, *p* = 0.001), CD41a^+^PMV (*r* = 0.327, *p* = 0.011), and CD45^+^LMV (*r* = 0.376, *p* = 0.003) levels (Figure 6). Elderly patients with OSA were then divided into 4 groups according to the number of comorbidities. We found that as comorbidities increased, AHI presented an increasing trend. Correspondingly, the MV levels showed an increasing trend with a number of comorbidities observed in patients. However, there were no significant differences between the 4 groups (*p* > 0.05; Figure 7).

Finally, we used multiple linear regression analysis to comprehensively analyze the effects of these factors on MV levels in elderly patients with OSA. The results showed that the BMI most significantly affected the levels of circulating MVs in elderly patients with OSA, when the influence of age, sex, and comorbidities were excluded (Tables 5, 6).

**Table 5:** Multivariate Regression Analysis of factors for circulating single-labeled MVs.

**Independent variables**	**Multivariate regression analysis**							
	**Annexin V**^****+****^**MV**		**CD144**^****+****^**EMV**		**CD41a**^****+****^**PMV**		**CD45**^****+****^**LMV**	
	**β**	**95% CI**	**β**	**95% CI**	**β**	**95% CI**	**β**	**95% CI**
Gender	0.037	−56.647 ~ 75.24	0.125	−59.259 ~ 183.567	−0.027	−969.745 ~ 776.629	0.096	−150.445 ~ 334.525
Age	0.117	−1.605 ~ 3.72	0.220	−0.938 ~ 8.867	−0.141	−53.784 ~ 16.727	0.094	−6.552 ~ 13.029
BMI	0.227	−1.313 ~ 10.78	0.453	7.673 ~ 29.938^*^	0.376	33.7 ~ 193.829^*^	0.362	6.586 ~ 51.054^*^
Comorbidities	−0.097	−39.302 ~ 18.431	0.087	−34.529 ~ 71.768	0.122	−191.101 ~ 573.37	0.075	−75.162 ~ 137.133

* *P <0.05*.

**Table 6:** Multivariate Regression Analysis of factors for circulating dual-labeled MVs.

**Independent variables**	**Multivariate Regression Analysis**					
	**Annexin V**^****+****^**CD144**^****+****^**EMV**		**Annexin V**^****+****^**CD41a**^****+****^**PMV**		**Annexin V**^****+****^**CD45**^****+****^**LMV**	
	**β**	**95% CI**	**β**	**95% CI**	**β**	**95% CI**
Age	−0.014	−6.541 ~ 5.882	−0.062	−10.466 ~ 6.263	−0.042	−6.036 ~ 4.281
Gender	−0.188	−0.414 ~ 0.088	−0.212	−0.596 ~ 0.08	−0.214	−0.368 ~ 0.048
BMI	0.167	−0.236 ~ 0.903	0.254	−0.055 ~ 1.479	0.249	−0.043 ~ 0.903
Comorbidities	0.020	−2.509 ~ 2.929	0.087	−2.395 ~ 4.929	0.061	−1.719 ~ 2.798

## Discussion

In this study, concentrations of circulating MVs in elderly patients with OSA were measured for the first time. To provide a basis for future research, we used single-labeled and dual-labeled detection methods to define and measure the MVs. At the same time, we analyzed correlations between the MV concentrations and the severity of OSA, and factors including age, obesity, and comorbidities, which had the potential to affect concentrations of MVs in elderly patients with OSA.

Although the prevalence of OSA is higher in the older populations than in the younger ones (Malhotra et al., 2006), it does not increase with age (Xun et al., 2019). Our study revealed that the prevalence of OSA in the super-elderly (>80 years) was lower than that of the elderly (60–80 years old). This phenomenon may be due to the premature death of older patients with OSA caused by various comorbidities, whereas the surviving elderly either do not have or have only mild OSA. Compared with the N-OSA group, the OSA group had a higher rate of obesity, blood lipids, and prevalence of hyperlipidemia, indicating that even in the elderly population, obesity, and hyperlipidemia remain as risk factors for the occurrence of OSA (Senaratna et al., 2017). There was no significant difference between sex observed between the elderly OSA group and the elderly N-OSA group, since there were no significant differences in the prevalence rates of OSA when postmenopausal women and men were compared (Hirotsu et al., 2017). The OSA and N-OSA groups differed according to AHI, ODI, SpO_2min_, SpO_2mean_, AHT_max_, and snore among the recorded indicators of PSG. This showed that it is appropriate to use these indicators to characterize the severity of OSA in the elderly. Other indicators, including AHT_mean_, MAI, TST, and sleep efficiency, may not be effective for reflecting the severity of OSA in the elderly.

Our study found that single-labeled annexin V^+^MVs, CD144^+^EMVs, CD41a^+^PMVs, and CD45^+^LMVs were elevated in elderly patients with OSA. A previous study found that patients with minimally symptomatic OSA had higher concentrations of annexin V^+^MVs vs. patients without OSA (Ayers et al., 2009), which is consistent with our research. There are several possible links between OSA and the creation of annexin V^+^MVs. For example, intermittent hypoxia could induce apoptosis, aggravate the vascular endothelial injury, and promote the expression of adhesion molecules (Shi et al., 2020; Wu et al., 2020).

Several studies have demonstrated an important role for EMVs in OSA. Some studies found that EMVs were increased in patients with OSA, and significant elevations in the concentrations of EMVs were reversed with continuous positive airway pressure (CPAP) treatment (Jelic et al., 2009; Yun et al., 2010). Ayers et al. (2013) further demonstrated that short-term withdrawal of the CPAP therapy led to a significant increase in EMVs, suggesting that the EMV formation may be causally linked to OSA and may promote endothelial activation and apoptosis. Tuleta et al. (2014) demonstrated that intermittent hypoxia contributed to endothelial dysfunction *via* a local increase in reactive oxygen species and reduction of the peripheral repair capacity, which increased the concentration of EMVs. Further experiments demonstrated that intermittent hypoxia impaired endothelial function and integrity in early pre-atherosclerosis. In contrast, Priou et al. (2010) and Tual-Chalot et al. (2012) reported no increase in EMV concentrations in OSA, probably because they selected CD146 as an EMV marker. The CD146 has been shown to be less adequate for investigating vascular damage than other markers, such as CD31 and CD144. In addition, Ayers et al. (2009) did not observe an increase in CD31^+^CD41^−^EMVs in OSA because they selected mild OSA as the case group.

The present study revealed that concentrations of CD41a^+^PMVs were significantly higher in patients with OSA than in patients without OSA. Our study is consistent with the studies conducted by Ayers et al. (2009), Bikov et al. (2017), and Maruyama et al. (2012). The PMVs possess a wide range of properties, including prothrombotic, proatherogenic, proinflammatory, immunomodulatory, and even anticoagulant activities (Rosinska et al., 2017). Studies have shown that PMVs can cause endothelial dysfunction by inducing the production of reactive oxygen species, reducing the concentration of nitric oxide, and inhibiting the activities of endothelial nitric oxide synthase and superoxide dismutase (Zhang et al., 2018). However, Geiser et al. (2002) found no difference in PMV concentrations between patients with and without OSA. Possible explanations for this negative finding may be due to the small sample size assessed in the study, and the techniques used to detect PMVs.

We found that the concentrations of CD45^+^LMVs were higher in the OSA group than in the N-OSA group. A study found that even in minimally symptomatic adults with OSA, concentrations of CD45^+^LMV were higher than those in control participants (Ayers et al., 2009). Priou et al. (2010) showed an increased concentration of CD66b^+^ LMVs and CD61L^+^LMVs in patients with OSA having nocturnal desaturations vs. control participants. These studies provided strong evidence for the fact that there is a close relationship between the LMV concentration and OSA. The LMVs modify the endothelial function and promote the recruitment of inflammatory cells to the vascular wall, which are necessary processes for the progression of the atherosclerotic lesions (Angelillo-Scherrer, 2012).

For the dual-labeled MV types, we found that the concentrations of annexin V^+^CD144^+^EMVs alone increased significantly in the OSA group vs. N-OSA group. This was not entirely consistent with the results of a previous study on children in which all MV types were found to be increased, including annexin V^+^CD31^+^/CD42b^−^EMVs, annexin V^+^ CD62E^+^/CD42b^−^EMVs, annexin V^+^CD41a^+^PMVs, annexin V^+^CD11b^+^LMVs, and annexin V^+^CD45^+^LMVs, which were elevated in patients with OSA (Kim et al., 2011). This may be related to the research population assessed, since there are fewer confounding and interfering factors in children with OSA. We did not observe an increase in annexin V^+^CD41a^+^PMVs and annexin V^+^CD45^+^LMV in elderly patients with OSA. The reason for this may be that intermittent hypoxia-activated platelets and leukocytes did not cause apoptosis, since annexin V is a marker that binds with PS and reflects apoptosis. Studies also suggested that the fraction of MVs that bind annexin V varies widely, from a few percent for MVs from activated cells to 80% for MVs from apoptotic cells (Horstman et al., 2004). In addition, the expressed dual-labeled MVs were low in our study, which might have affected the significance of the statistical differences. Our research suggests that the different labeling detection methods, both single-and dual-labeling, may have an impact on the results. However, it appears that the dual-labeled MVs are more selective than the single-labeled MVs. Future experiments should use either single- or dual-labeling methods according to the specific experimental requirements.

We analyzed the correlation between MV types and parameters reflecting the severity of OSA. This work not only assessed the correlation between MV levels and OSA severity in the elderly, but also supplemented other studies that assessed single indicators. Previous studies generally focused on the correlation between AHI and a certain kind of MV type, whereas we analyzed the correlation between important indicators reflecting the severity of OSA in the elderly and various MV types. We found a positive correlation between the most measured MVs and AHI, ODI, SpO_2min_, and SpO_2mean_, which was consistent with the results of Maruyama et al. (2012). However, we did not observe a correlation between MV levels and either snore or AHT. Therefore, the increased MV concentration may not be related to temporary breathing and snoring. Interestingly, it may be due to exposure to repeated hypoxia during sleep.

Finally, we analyzed the principal factors that affect levels of circulating MVs in elderly patients with OSA. Although several studies have shown that the concentrations of circulating MVs in normal elderly people are higher than those in young people (Arauna et al., 2020), in elderly patients with OSA, increasing age was not positively correlated with higher concentrations of MVs. In elderly patients with OSA, AHI decreased as age increased (Hiestand et al., 2006), which is consistent with observed trends, which showed a positive correlation between the MV concentration and AHI. In elderly patients with OSA, the higher the BMI, the higher the MV concentration, which is consistent with the positive correlation between AHI and obesity. Moreover, an increased incidence of cardiovascular, cerebrovascular, and metabolic diseases was observed in elderly patients with OSA. The existence of comorbidities also tended to be associated with increased concentrations of MVs. However, the effect of comorbidities on circulating MVs requires further verification using a larger sample size. In addition, the severity of comorbidities and the use of drugs were not identical between groups assessed, which may be the reason for the observation of no significant differences. Multiple linear regression analysis showed that obesity (evaluated *via* BMI) was the main factor that affected the MV levels in elderly patients with OSA after controlling for other factors. This suggests that obesity increases the concentration of MVs in elderly patients with OSA, such that elderly patients with OSA would likely benefit from weight loss.

Our study had some limitations. First, contributions of other subtypes of EMVs, including CD31^+^EMVs, CD146^+^EMVs, and CD62E^+^EMVs, to OSA should also be fully explored. Second, a large, long-term follow-up study will be needed to enhance the power of the study and validate our results. Third, we did not further investigate the mechanism underlying the increase in OSA and MV concentration, which may involve oxidative stress and inflammatory factors.

## Conclusion

In the current study, we found that the concentrations of circulating MVs were elevated in elderly patients with OSA compared to the concentrations in those without OSA. This increase was observed *via* the detection of both single-labeled MVs (annexin V^+^MVs, CD144^+^EMVs, CD41a^+^PMVs, and CD45^+^LMVs), and dual-labeled MVs (annexin V^+^CD144^+^EMVs). Furthermore, increased concentrations of MVs were positively correlated with OSA severity (AHI, ODI, and SPO_2min_); as OSA severity increased, MV concentrations also increased. Finally, MV concentrations in elderly patients with OSA were affected by obesity to some extent. In addition, age and comorbidities may be associated with the MV levels, but the correlation between MV levels and age or comorbidities was not significant. In short, concentrations of circulating MVs in elderly patients with OSA depend on labeling methods and the severity of OSA and are also affected by obesity. The effects of age and comorbidities on circulating MVs require further verification using a larger sample size.

## Data Availability Statement

The raw data supporting the conclusions of this article will be made available by the authors, without undue reservation.

## Ethics Statement

The studies involving human participants were reviewed and approved by ethical committee of Tianjin Medical University General Hospital. The patients/participants provided their written informed consent to participate in this study.

## Author Contributions

JT designed the study, edited the statistical analyses, and revised the manuscript. HX collected the data, edited the statistical analyses, and revised the manuscript. SS contributed to statistical analyses and interpretation of data. JL contributed to statistical analyses and edited the manuscript. YM contributed to manuscript editing. QZ designed the study and edited the manuscript. All authors approved the manuscript and are accountable for the final version of the manuscript.

## Conflict of Interest

The authors declare that the research was conducted in the absence of any commercial or financial relationships that could be construed as a potential conflict of interest.
